# Comparative Analysis of Physicochemical and Functional Properties of Pectin from Extracted Dragon Fruit Waste by Different Techniques

**DOI:** 10.3390/polym16081097

**Published:** 2024-04-15

**Authors:** Huimin Du, Ibukunoluwa Fola Olawuyi, Nurul Saadah Said, Won-Young Lee

**Affiliations:** 1 School of Food Science and Technology, Kyungpook National University, Daegu 41566, Republic of Korea; du19981212@knu.ac.kr (H.D.); ifolawuyi@knu.ac.kr (I.F.O.); nurulsaadah.said@gmail.com (N.S.S.); 2 Research Institute of Tailored Food Technology, Kyungpook National University, Daegu 41566, Republic of Korea

**Keywords:** dragon fruit peel, waste utilization, pectin, extraction method, structure–function

## Abstract

Dragon fruit peel, often discarded, is a valuable source of commercial pectin. This study investigates different extraction methods, including cold-water (CW), hot-water (HW), ultrasound (US), and novel enzyme extraction (xylanase: EZX), to extract pectins from dragon fruit peel and compare their characteristics. The pectin yield ranged from 10.93% to 20.22%, with significant variations in physicochemical properties across methods (*p* < 0.05). FTIR analysis revealed that extraction methods did not alter the primary structural configuration of the pectins. However, molecular weights (Mws) varied significantly, from 0.84 to 1.21 × 10^3^ kDa, and the degree of esterification varied from 46.82% to 51.79% (*p* < 0.05). Monosaccharide analysis identified both homogalacturonan (HG) and rhamnogalacturonan-I (RG-I) pectic configurations in all pectins, predominantly comprising galacturonic acid (77.21–83.12 %mol) and rhamnose (8.11–9.51 %mol), alongside minor side-chain sugars. These properties significantly influenced pectin functionalities. In the aqueous state, a higher Mw impacted viscosity and emulsification performance, while a lower Mw enhanced antioxidant activities and promoted the prebiotic function of pectin (*Lactis brevies* growth). This study highlights the impact of extraction methods on dragon fruit peel pectin functionalities and their structure–function relationship, providing valuable insights into predicting dragon fruit peel’s potential as a food-grade ingredient in various products.

## 1. Introduction

Dragon fruit, a member of the cactus family, is extensively cultivated and consumed in subtropical regions of South America, Southeast Asia, and China. In recent times, temperate countries, including South Korea, have significantly increased their efforts to cultivate subtropical fruits, resulting in successful dragon fruit cultivation in areas such as Jeju and Tongyeong [1]. The peel of dragon fruit, which constitutes 30% of the fruit, is often discarded as waste in the food-processing industry, leading to unexplored potential value, economic inefficiencies, and environmental challenges [2]. As a result, significant research efforts have been directed at repurposing these by-products, with recent studies predominantly concentrating on the extraction of pectin from dragon fruit peels [3,4,5,6,7]. The dragon fruit peel, characterized by a high moisture content exceeding 90%, constitutes a significant source of pectin [8,9]. The proximate composition of the peel of dragon fruit comprises 64.14% to 72.72% carbohydrates, 15.05% to 21.66% ash, 3.44% to 9.27% protein, and 0.40% to 1.66% fat [10]. Its distinctive purplish-red hue is attributed to betacyanin, a natural water-soluble nitrogen-containing pigment originating from tyrosine in fruits and vegetables, renowned for its noteworthy anti-inflammatory and antioxidant properties [11]. Furthermore, owing to its vibrant coloration, betacyanin serves as an important natural food colorant widely employed in food-processing applications [12].

Pectin obtained from dragon fruit peel is regarded as a versatile ingredient with various applications in the food industry, functioning as a thickener, stabilizer, and emulsifier and a food fortificant in various food products such as jam, jelly, sauces, and ice cream [13,14]. Moreover, dragon fruit peel pectin has been used to develop biodegradable films as potential food-packaging materials [15]. In terms of health benefits, it has been documented to exhibit biological activities such as anti-inflammatory, antioxidant, and prebiotic properties [16]. However, the functionalities of pectin have been linked to its structural and physicochemical composition, which are determined by factors including their sources and especially the extraction methods employed [17,18,19,20]. Furthermore, the choice of extraction method is important for achieving high pectin yield and quality [21]. Thus, it is believed that different extraction methods could significantly impact desirable functionalities in dragon fruit peel pectin. In most studies, heat extraction methods with inorganic chemical acid solvents are employed in pectin extraction from dragon fruit peel [21]. While this method results in high pectin yield (14.11–19.48%) [22,23], the use of chemical solvents like sulfuric and citric acid raises environmental concerns and may affect consumer preference [24,25]. This has prompted the exploration of alternative extraction methods that support the use of less toxic and hazardous chemicals.

In recent years, novel methods, such as enzyme-, microwave-, and ultrasonic-assisted techniques, have been shown to improve pectin yield and quality [7,26]. Ultrasound-assisted extraction utilizes ultrasonic waves to improve solvent–solute contact, thus enhancing extraction efficiency while reducing solvent use and allowing for extraction at lower temperatures [2]. For instance, Wang et al. [9] observed a 16.34% increase in pectin yield at a lower extraction temperature (13.3 °C) and a reduced extraction time (37.78%) compared with conventional hot water extraction. Enzymatic extraction is another eco-friendly approach involving using enzymes under specific conditions to selectively depolymerize cell wall components (pectin, cellulose, and hemicelluloses) without harsh chemicals [27]. In the cell wall of dragon fruit peel, the pectin is strongly attached to hemicelluloses (xyloglucans and xylans) and cellulose, limiting its efficient extraction [28]. The xylanase enzyme, which catalyzes xylan hydrolysis, can disrupt the cell wall integrity and offer the targeted cleavage of these hemicelluloses into soluble fragments, thereby facilitating the release of bound pectin substance [29,30]. However, there is limited information on the characteristics and functionalities of pectin obtained from dragon fruit peel by enzymatic extraction. Therefore, this study comparatively examines the impact of hot- and cold-water extraction, ultrasonic-assisted extraction, and a novel enzymatic approach using xylanase on the characteristics, functionalities, and potential applications of pectin from dragon fruit peel.

## 2. Materials and Methods

### 2.1. Materials

Fresh dragon fruit (*Hylocereus undatus*) was obtained from a local farm in the Republic of Korea. To obtain the dried peel powder, the peel was separated from the fruit pulp manually with a knife, cut into small fragments, and then dried for 48 h at 45 °C in a hot air dryer. Subsequently, the dried pieces were ground into a powder using a grinder and stored in a tightly sealable bag at −18 °C until further use. All chemical solvents used were of analytical grade and obtained from Duksan Chemicals (Ansan, Republic of Korea). The enzyme Shearzye^®^ 500 L, which contains xylanase (endo-1,4-) with a declared activity of 500 FXU-S/g, was obtained from Novozymes, Bagsvaerd, Denmark.

### 2.2. Pectin Extraction

Four different extraction methods were used to extract pectin from the peel of dragon fruit, namely, cold-water extraction (CW), hot-water extraction (HW), ultrasonic-assisted extraction (US), and enzyme-assisted extraction using xylanase (EZX), as described below. After the completion of extraction across all five methods, the resultant supernatants were centrifuged at 4000 rpm for 10 min using a SUPRA-22K centrifuge, Hanil Science Industrial, Incheon, Republic of Korea). To precipitate the pectin, the supernatants were mixed with 95% ethanol (1:2 *v*/*v*) and kept overnight at 5 °C. Then, the precipitated pectin was separated by centrifuging the mixture for 10 min at 4000 rpm. This precipitate was subjected to two rounds of washing using 95% ethanol, after which, freeze-drying was performed. The obtained powder was stored in a sealable bag at −18 °C prior to analysis. Protein analysis was performed using the Bradford assay method.

#### 2.2.1. Cold-Water Extraction (CW)

The pectin extraction was prepared by mixing 10 g (*w*/*v*) of dried dragon fruit peel powder with 300 mL of distilled water and subjecting it to a temperature of 5 °C for 48 h [31]. 

#### 2.2.2. Hot-Water Extraction (HW)

The pectin extraction was prepared by mixing 10 g (*w*/*v*) of dried dragon fruit peel powder mixed with 300 mL of distilled water in a glass flask. The mixture was placed in a heating water bath at an elevated temperature of 50 °C for 2 h.

#### 2.2.3. Ultrasonic-Assisted Extraction (US)

For US extraction, 10 g (*w*/*v*) of dried dragon fruit peel powder was mixed with 300 mL in a glass flask and placed in an ultrasonic machine (KHC-1SUMP, Kyung il Ultrasonic, Ansan, Republic of Korea), with working conditions of 600 W and 100 kHz at 50 °C for 30 min [32].

#### 2.2.4. Enzyme-Assisted Extraction (EZX)

For EZX, 10 g of dried dragon fruit peel powder was dissolved in 300 mL of 0.1 M sodium acetate buffer (pH 4.5) containing 1 mL of xylanase enzyme. The mixture was then placed in a shaking incubator at 50 °C for 2 h. The enzyme was deactivated by heating the mixture at 100 °C for 5 min, and pectin was precipitated as described in Section 2.2. The buffer and pH used were specified as the optimal conditions for the enzyme by the manufacturer.

### 2.3. Color Analysis

The L*, a*, and b* values of the pectin samples were measured using a Chroma Meter colorimeter (CR-300, Minolta Co., Osaka, Japan) in accordance with the CIE color measurement system [33].

### 2.4. Monosaccharide Composition of Pectins

High-performance liquid chromatography (HPLC) (JASCO International Co., Ltd., Tokyo, Japan) coupled with an Athena C18 reverse-phase column (250 mm × 4.6 mm; 5 µm) was used to analyze the monosaccharide composition of the pectin as described by Olawuyi and Lee [32]. Initially, 4 mL of 2 M trifluoroacetic acid (TFA) was used to dissolve 0.01 g of each pectin sample in a glass vial. Afterward, the vial was sealed and autoclaved for 60 min at 121 °C. TFA was evaporated with the aid of methanol using a rotary evaporator, and the concentrated sample was further dissolved in 4 mL of HPLC-grade water. An aliquot (0.5 mL) of the hydrolysate was mixed with 0.5 mL of 0.3 M NaOH and 0.5 mL of 0.5 M PMP in methanol. The mixture underwent a reaction for 1 h at 70 °C, after which, it was cooled to room temperature and neutralized with 0.5 mL of 0.3 M HCl. The resulting solution was then extracted three times with chloroform. The derivatized hydrolysate was filtered through a 0.45 µm membrane, and the monosaccharide composition was measured and quantified using the respective standard calibration curves.

### 2.5. Molecular Weight of Pectins

The determination of pectin molecular weight (Mw) and distribution followed the procedure outlined in our prior studies [34,35]. Gel permeation chromatography, utilizing a refractive index detector (Thermo Dionex, HPLC Ultimate3000 RI System, JASCO International Co., Ltd., Tokyo, Japan) equipped with Waters Ultrahydrogel columns (120, 500, and 1000), was employed. Pectin solutions (5 mg/mL) were prepared in 0.1% sodium azide and filtered before injection into the system. Elution was conducted at 40 °C with 0.1 M sodium azide as the mobile phase at a flow rate of 1 mL/min. Data analysis utilized the Chromeleon Version 6.8 Extension-pak software, with pullulan serving as a standard for quantification.

### 2.6. FTIR Structural Analysis and DE Quantification

Dragon fruit peel pectin (2 mg) was mixed with 100 mg of KBr, and spectra were measured from 4000 to 400 cm^−1^ at a resolution of 4 cm^−1^ using an FTIR spectrophotometer, Frontier, Billerica, MA, USA [32]. The degree of esterification (DE) was calculated by comparing the FTIR peak area values of the free carboxyl groups (1630 cm^−1^) and the esterified groups (1740 cm^−1^) using the equation formulated by [36].

(1) DE=124.7×R+2.201

(2) R=A1740/(A1740+A1630)×100

### 2.7. Viscosity of Pectin Solutions

A DV-II + PRO viscometer (Brookfield Engineering Laboratories, Inc., Middleboro, MA, USA) with an attached spindle (No. 42) was used to measure apparent viscosity. The flow behavior was measured using a 1% (*w*/*v*) pectin solution [32]. To demonstrate the flow behavior, readings for apparent viscosity (η) were taken at different shear rates (γ) at 25 °C and plotted as a line graph. The flow behavior of pectin in aqueous solution was predicted using the Power Law Model below:

(3) $$\eta =K\gamma^{n-1}$$

where η is the apparent viscosity, *K* is the flow consistency index, γ is the shear rate, and *n* is the flow behavior index.

### 2.8. Emulsifying Properties of Pectin Solutions

Pectin powder was dissolved in 0.1 M sodium azide buffer to prepare a pectin solution (1% *w*/*v*). In order to produce an oil-in-water emulsion, 10 mL of pectin solution and 10 mL of soybean oil were mixed and homogenized (PT-1200C, Kinematica AG, Littau, Switzerland) for 1 min at 20,000 rpm [32]. To determine the emulsifying capacity (EC), the prepared emulsions were centrifuged for 10 min at 4000 rpm. The emulsion stability (ES) was assessed by placing the prepared emulsions in a hot water bath for 30 min at 80 °C, followed by centrifugation for 10 min at 4000 rpm. The percentages of EC and ES were calculated using the following equation:

(4) EC (%)=(Ev/Tv)×100

where Ev is the volume of the emulsified layer, and Tv is the total volume of the emulsion phase.

(5) ES (%)=(Fev/Iev)×100

where Fev is the final emulsion phase volume following treatment in a water bath, and Iev is the initial emulsion layer [37].

### 2.9. Betacyanin Content of Pectins

Betacyanin extraction was carried out by mixing 0.05 g of pectin powder into 2 mL of 80% aqueous MeOH acidified with 5% formic acid (*v*/*v*) under stirring for 2 h at 200 rpm [38]. Thereafter, a clear supernatant was obtained by centrifugation (10 min at 4000 rpm), and the absorbance was measured at 536 nm using a UV spectrophotometer (UV-2500, Shimadzu Corporation, Kyoto, Japan). The betacyanin concentration (BC) (mg/L) in the pectin extracts was quantified by the following Equation (6) [39]

(6) $$\mathrm{BC}=(A\times Df\times Mw\times 1000)/(E_{\%}\times L)$$

where E_%_ is the betalain’s extinction coefficient, A is the absorbance at 536 nm, Df is the dilution factor, Mw is the molecular weights of betacyanin, and L is the pathlength of the (1 cm) cuvette.

### 2.10. Antioxidant Activity of Pectins

The antioxidant activity of pectin was determined using ABTS radical scavenging assays, and ABTS reagent preparation and reaction followed the method described by Zhang et al. [40]. Briefly, 50 µL of pectin solution at 4 different concentrations (5 to 20 mg/mL) were added to 950 µL of ABTS solution. After reacting in the dark for 30 min, the absorbance was measured at 734 nm using ethanol as the blank. The ABTS radical scavenging activity (%) was calculated using Equation (7).

(7) $$\mathrm{ABTS}\;(\%)=(1-A_{1}/A_{0})\times 100$$

where A_1_ and A_0_ are the absorbances of the sample and blank, respectively.

### 2.11. Prebiotic Property of Pectins

The prebiotic properties of the pectin samples were examined using *B. animalis* subsp. *Lactis brevis*. For this experiment, pectin (1%) was dissolved in a sterile 0.1% saline solution, and MRS broth was used as a culture liquid medium. In total, 1 mL of pectin solution was mixed with 8.9 mL of MRS broth and 0.1 mL of pre-cultured *Lactis brevis* (10^4^ CFU/mL) and then placed in a shaking incubator at 37 °C for 12 h. Saline solution was used in place of pectin solution, serving as a control to compare the growth of the culture. After incubation, cells were harvested by centrifugation, washed with saline solution, redissolved in 10 mL of saline solution, and serially diluted for colony count using the plate assay method with MRS agar plates. The colony count on the plates was recorded and denoted as CFU/mL after 24 h at 37 °C in an anaerobic incubator [41].

### 2.12. Statistical Analysis

The results are presented as mean ± standard deviation, and statistical analyses were performed using SPSS software v.20 (SPSS Inc., Chicago, IL, USA). One-way analysis of variance (ANOVA) followed by Tukey’s post hoc tests were employed to compare the mean values, with a significance level set at *p* < 0.05.

## 3. Results and Discussion

### 3.1. Yield and Composition of Pectins

The yields and compositions of the pectin extracted using the four different extraction methods are demonstrated in Table 1. The pectin extraction yield was observed to significantly differ (*p* < 0.05) according to extraction methods, with enzyme-assisted extraction having the highest value (EZX: 20.22%), followed by US (15.17%), HW (15.03%), and CW (10.93%). The xylanase enzyme’s ability to cleave xylan and glucoxylans in the plant tissue could have increased the likelihood of the selective release of pectin from residual cell wall polymers, leading to a higher yield [42]. The reduced solubility of pectin at lower temperatures in cold-water extraction (CW) can limit its extraction efficiency [43], whereas higher temperatures facilitate both cell wall disruption and enhanced pectin release [44], thereby increasing the extraction yield. Moreover, higher yields were observed for both the hot-water (HW) and ultrasonic (US) methods compared with CW. This may be attributed to the high-temperature treatment involved in both HW and US methods, playing a significant role in the extraction, dissolution, and degradation of pectin [45]. The elevated temperature, combined with ultrasonic cavitation, effectively disrupts intermolecular and intramolecular covalent crosslinking in the cell wall matrix, easing the release of water-soluble pectin components into the plant matrix [46]. Thus, the US method achieved higher yields compared with those of HW in a shorter extraction time.

All pectin samples contained low protein content (3.37–4.56%) and can be considered pure pectin ingredients for food products, in accordance with the protein threshold (15.6%) recommended by the FAO [47]. 

The monosaccharide analysis and sugar molar ratios presented in Table 2 reveal that the extracted pectin is a polysaccharide complex with a main chain consisting of galacturonic acid (GalA) and rhamnose and minor sugars such as mannose, glucose (Glu), galactose (Gal), and arabinose (Ara). The pectin extracted by US showed the highest GalA content (83.12%) compared with the 77%–78% observed for samples from other extraction methods (*p* < 0.05). This study observed higher GalA content than those in other reported studies for dragon fruit pectin extraction, such as 30.58–56.18% [3], 39.11% [48], and 59.73–69.68% [49]. The GalA content in all samples exceeded 65%, indicating that these extracted pectins could be commercialized in the market, aligning with the commercial pectin benchmark recommended by Guandalini et al. [50]. Rha, Gal, and Glu had sugar compositions of 8.76–9.51%, 3.68–4.71%, and 2.70–5.51%, respectively. However, Man and Ara showed lower concentrations of dragon fruit pectin (0.59–1.63%; 0.93–3.86%) compared with other sugar compositions. Table 1 illustrates that all the extracted pectins exhibited a Rha/GalA ratio within a range of 0.10–0.12, classifying them as RG-I pectins. This aligns with the established threshold (0.05–1.00) associated with RG-I pectin classification [51]. The pectin obtained from different sources varies based on major domains (homogalacturonan, HG; rhamnogalacturonan-I, RG-I), and the extent of esterification determines key functional properties such as gelling, viscosity induction, and stabilizing [52]. Hence, based on these findings, it can be inferred that the monomeric composition of pectin can be tailored by extraction with different methods to achieve different functionalities.

### 3.2. Color and Betacyanin Content of Pectins

The color of extracted pectin was significantly influenced by different extraction conditions, directly impacting the visual appearance of the final product. The color analysis, represented by L*, a*, and b*, is presented in Table 1. EZX pectin exhibited the highest L* value (69.08), indicating a brighter appearance. Conversely, the CW method, without heat treatment, had the highest a* value, and US and HW resulted in progressively lower a* values (CW > US > HW), indicating a decrease in red color intensity, attributed to betacyanin breakdown or separation. Enzyme-assisted extraction showed a diminished red hue compared with the non-enzymatic treatment, suggesting enzymatic treatment may have degraded or altered the betacyanin during the extraction process [53]. Moreover, the b* value reflecting yellowness was higher in EZX compared with other pectins.

The betacyanin contents of all samples are presented in Table 1, as calculated using Equation (6). Betacyanin, a natural pigment imparting a purplish-red color, serves as the primary pigment component in dragon fruit peel. However, this pigment is inherently unstable and susceptible to degradation from factors such as heat, oxygen, light, pH, and moisture [54]. The results denote the betacyanin contents, corresponding to the a* value in color analysis (Table 1). CW exhibited the highest betacyanin content (50.65 mg/L) and exhibited the highest a* value. This can likely be attributed to the enhanced stability of betacyanin in low-temperature extraction [55]. The betacyanin contents of US (43.49 mg/L) are higher than HW (31.01 mg/L). This difference may arise from the fact that, despite both being extracted at 50 °C, the US sample had a shorter extraction time than HW, allowing it to retain more betacyanin. On the other hand, pectins obtained through enzyme extraction (EZX) exhibited the lowest betacyanin contents (20.74 mg/L; 7.89 mg/L). Enzymes release betacyanin from cell walls by degrading the cell wall components [56]. During the pectin precipitation by ethanol, the betacyanin released during the enzyme treatment might have dissolved into the ethanol solvent, resulting in a higher loss of betacyanin in the EZX pectin.

### 3.3. Molecular Weight of Pectins

Molecular weight is one of the most important attributes determining the potential applications of pectin in aqueous solutions [57]. CW pectin has a significantly higher Mw (1.21 × 10^3^ kDa) compared with HW, US, and EZX, with Mw values of 1.01 × 10^3^ kDa, 0.97 × 10^3^ kDa, and 0.84 × 10^3^ kDa, respectively. The Mw of pectins in this study falls within a similar range for pectins (0.09–1.18 × 10^3^ kDa) extracted from dragon fruit peel in previous studies [21,48]. Notably, the observed range in this study surpassed the molecular weights of apple and citrus pectins recorded at lower values (0.144 × 10^3^ kDa and 0.138 × 10^3^ kDa, respectively [48]. A higher Mw in CW pectin may imply lesser molecular defragmentation at lower temperatures, whereas elevated temperatures used in HW and additional cavitational force in the US method can catalyze hydrolysis, leading to the fragmentation of the pectin molecule and a subsequent decline in molecular weight [58]. In addition, EZX, having the lowest molecular weight, could be attributed to the enzymatic cleavage of the molecular chains at different sites caused by xylanase, resulting in a greater reduction compared with other methods [37]. 

The molecular distribution result (Mw/Mn) indicates that EZX has a more widely distributed molecular configuration (13.04) compared with other methods (4.19–4.81) [59]. The higher value observed can be attributed to enzymatic degradation resulting in fragments with a broader range of molecular weights within the sample. In contrast, the lower Mw/Mn value of pectins obtained from other methods suggests a more uniform molecular distribution, and the values observed in this study align with the results (1.21–8.12) reported for the molecular distribution of dragon fruit peel pectin [14,48].

### 3.4. Structural Analysis and DE Quantification of Pectins

**Figure 1:**
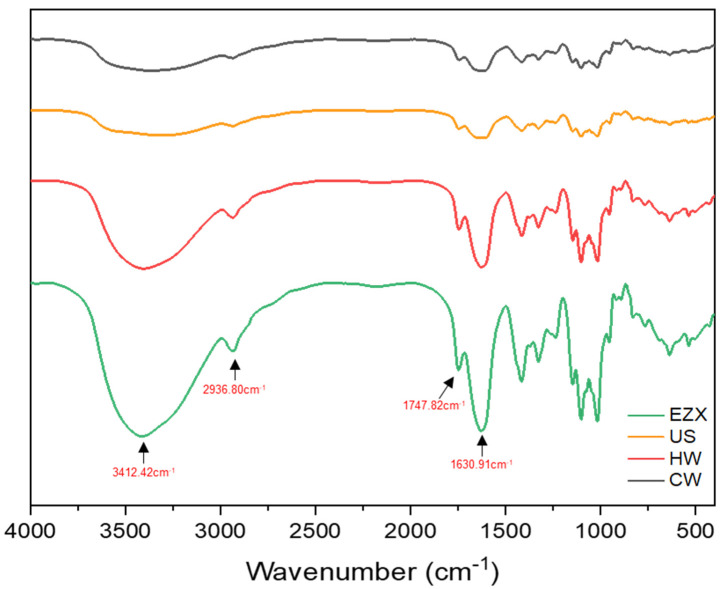
FTIR spectra of pectin. CW = cold-water extraction; HW = hot-water extraction; US = ultrasound-assisted extraction; EZX = Enzyme-assisted extraction (xylanase).

FTIR spectra (Figure 1) revealed that all pectins had similar structural characteristics, exhibiting the same FTIR peak pattern, indicating that the extraction method did not influence the primary structural conformation [60]. The strong band detected around the 3412 cm^−1^ region can be attributed to O–H stretching arising from intermolecular and intramolecular hydrogen bonding of sugars present in pectin [34]. The region near 2936 cm^−1^ signifies double-stacked stretching bands caused by C–H absorption, including pectin’s CH, CH_2_, and CH_3_ stretching and bending vibration of sugars and methyl ester of GalA [35,61]. The absorption peak at around 1747 cm^−1^ corresponds to the C=O stretching vibration of the methyl-esterified carbonyl group, and the strong peak around 1630 cm^−1^ signifies the COOH asymmetric bond [34].

The degree of esterification (DE) of pectin was estimated based on the ratio of esterified carboxylic groups to total carboxylic groups in the FTIR spectra, as calculated using Equations (1) and (2) [60,62]. Pectins are classified as high-methoxyl pectins (HMPs, DE ≥ 50%) and low-methoxyl pectins (LMPs, DE < 50%) [63]. The extracted pectins exhibited varying DE values, with the highest DE found in the US pectin (51.79%), followed by CW (50.88%), classifying them as high-methoxyl pectins (HMPs). Meanwhile, EZX (47.74%) and HW (46.82%), with lower DE values, could be classified as LMPs. The variations in DE observed here may be attributed to differences in the extraction process, that is, the heating time in HW and the specificity of enzymatic action in EZX methods. These differences in DE could equally translate to differences in the functional properties of the respective pectins, such as their gelling properties. For instance, US and CW, which are HMPs, could form a gel network in an acidic medium (below pH 3.6) in the presence of abundant sugar (more than 55%). In contrast, LMPs (HW and EZX) can gel in the presence of cations, such as calcium ions, under mild pH conditions (2.0 to 6.0), without the addition of sugar [63]. This property is particularly relevant in products like jams, jellies, and fruit preserves, where the desired texture and stability depend on the gelling capability of the pectin [64].

### 3.5. Viscosity of Pectin Solutions

**Figure 2:**
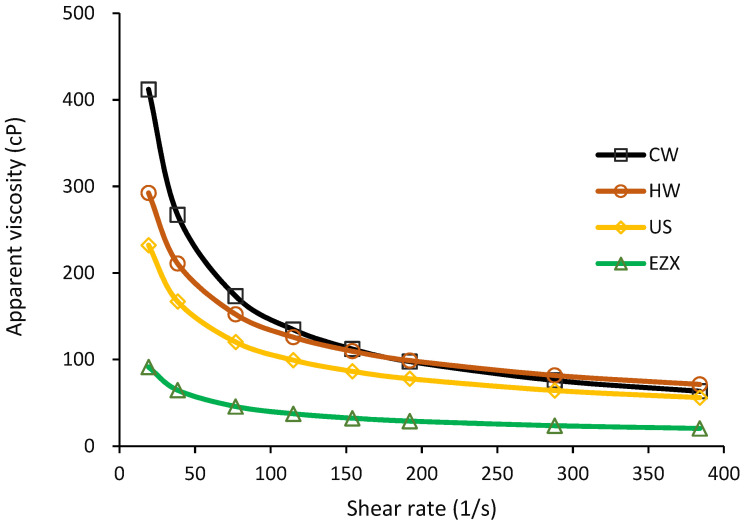
Viscosity of pectin solutions. CW = cold-water extraction; HW = hot-water extraction; US = ultrasound-assisted extraction; EZX = enzyme-assisted extraction (xylanase).

The apparent viscosity of the extracted pectins (Figure 2) was observed to correlate directly with their molecular weights (CW > HW > US > EZX). The rheological parameters, including the flow consistency index (*K*), the flow behavior index (*n*), and the correlation coefficients (*r*^2^) of the pectin solutions, are summarized in Table 3, as calculated using Equation (3). High *r*^2^ values (>0.99) indicate a good model fit. The observed shear-thinning behavior (*n* < 1.0) in the apparent viscosity with increasing shear rate confirms the pseudo-plastic property of pectin in aqueous solution [35]. This behavior is consistent with the decrease in flow resistance due to elevated shear rates [65], resulting from the reduction in intermolecular entanglement between pectin chains [66]. The observed viscosity behavior aligns with findings from a previous study on dragon fruit peel pectin [14] and pectins from other fruit sources, such as apples [67]. The detected consistency index (*K*) follows the order CW (2613.15), HW (1176.96), US (943.42), and EZX (400.41), aligning with their respective viscosities and molecular weights (Table 1). 

The viscosity property of pectin solutions is essential in applications where controlling viscosity is crucial for achieving the desired texture, stability, and consistency of the final product. Consequently, pectins with higher molecular weights are often considered to be more suitable for specific applications that require distinct viscosity characteristics.

### 3.6. Emulsifying Properties of Pectins

**Figure 3:**
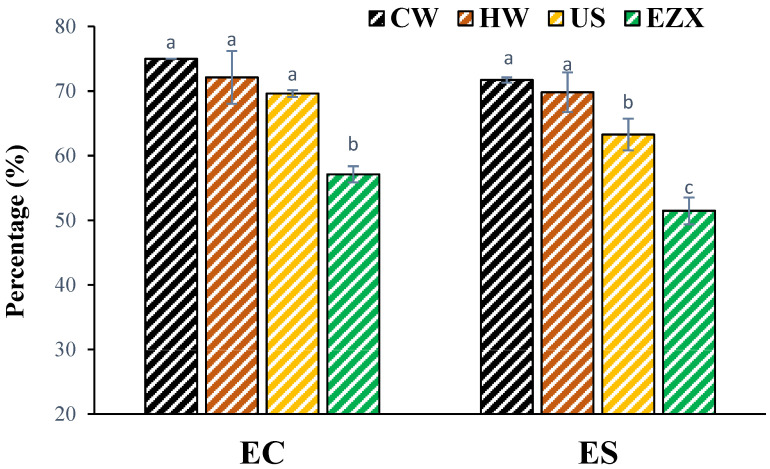
Emulsifying capacity (EC) and stability (ES) of pectin solutions in oil-in-water emulsion system (1:1). CW = cold-water extraction; HW = hot-water extraction; US = ultrasound-assisted extraction; EZX = enzyme-assisted extraction (Xylanase). Superscripts (a–c) indicate significant difference (*p* < 0.05).

The emulsifying properties of pectins represented by EC and ES values are presented in Figure 3, as calculated using Equations (4) and (5). CW pectin exhibited higher EC (75%) and ES (71.71%) values compared with the other samples, while EZX showed lower emulsifying properties (EC 57.13% and ES 51.47%) compared with the other pectin samples. The EC and ES trend was directly proportional to the molecular weight of the pectins. This aligns with research by Leroux et al. [68], emphasizing the significant impact of molecular weight on viscosity properties and emulsion stabilization. Higher molecular weights alongside longer chains in pectin have been reported to improve emulsion stabilization by better encapsulating and suspending oil droplets in the water phase, preventing coalescence and separation [68]. Also, the higher viscosity of the continuous phase in the emulsion system can slow down the movement of oil droplets, preventing the aggregation and coalescence of oil droplets [69]. In conclusion, pectins from CW, HW, and US showed better emulsifying capacities and stabilities, making them promising candidates for use as natural food emulsifiers compared with enzymatically extracted pectin. Additionally, similar to another study, no clear relationships were observed between the protein and DE and the emulsifying performance of pectin seen in some studies [34].

### 3.7. Antioxidant Activity of Pectins

**Figure 4:**
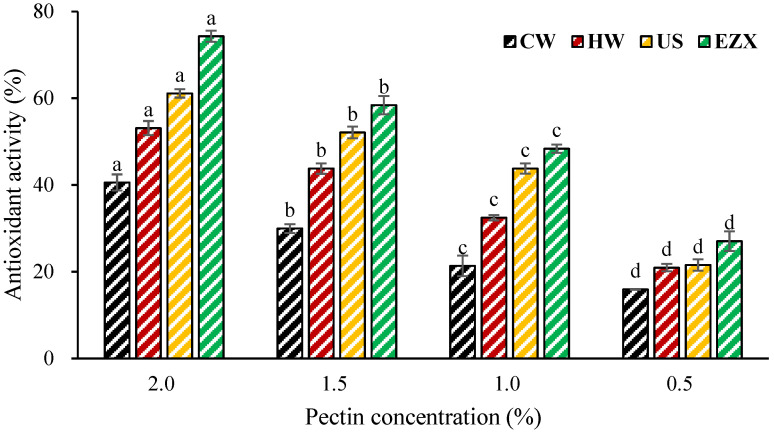
ABTS antioxidant activity of pectin solutions. CW = cold-water extraction; HW = hot-water extraction; US = ultrasound-assisted extraction; EZX = enzyme-assisted extraction (xylanase). Superscripts (a–d) indicate significant difference (*p* < 0.05).

The antioxidant activities of pectins were assessed using the ABTS radical scavenging assay, a widely utilized method for evaluating the total antioxidant activities of natural compounds [70]. This mechanism relies on the compound’s ability to scavenge free radicals in the ABTS reagents by donating hydrogen atoms, thus converting them into non-radical forms [51]. The antioxidant activity results are presented in Figure 4 as calculated using Equation (7). Among all the pectins, EZX pectin exhibited the highest ABTS radical scavenging activity (27.05–74.29%), followed by pectins from US (21.54–61.11%), HW (20.94–53.11%), and CW (15.93–40.58%). Correlation analysis revealed a positive relationship between the molecular weight (Mw) of the samples and the •OH radical scavenging activity. Both the ultrasound and enzymatic treatments resulted in pectins with lower Mws compared with other pectin samples. The decrease in pectin molecular weight exposes more hydroxyl groups to the solution, leading to a gradual increase in the scavenging rate of •OH radicals [71]. This aligns with prior research, suggesting that the reduction in pectin molecular weight due to ultrasound and enzymatic treatment [40,72] during the extraction process, combined with an increased number of –OH groups from degradation, significantly contributes to the enhancement of •OH radical scavenging activity [71]. Additionally, some factors, such as monosaccharides, and chemical properties, such as betacyanin contents [73], have been reported to impact the antioxidant activity of pectin, although no consistent trend was observed in this study.

### 3.8. Prebiotic Function of Pectin Solutions

**Figure 5:**
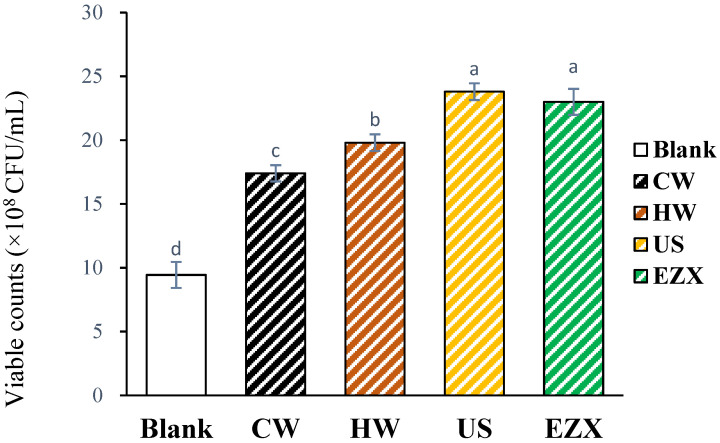
Viable counts of *Lactic Brevis* after incubation in liquid medium containing 1% pectin solution (*v*/*v*). Superscripts (a–d) indicate significant difference (*p* < 0.05). B = blank (0.1% saline solution), CW = cold-water extraction; HW = hot-water extraction; US = ultrasound-assisted extraction; EZX = enzyme-assisted extraction (xylanase).

Figure 5 illustrates the prebiotic properties of the pectin samples, indicating their positive impact on the proliferation of *Lactis brevis*. The probiotic potential of pectin may stem from the RG-I pattern of its structure [74]. Previous studies by Gamonpilas et al. [74] and Yeung et al. [75] have suggested that pectin with a higher RG-I region is more readily utilized by bacteria. According to the results, *Lactis brevis* exhibited higher growth after incubation in a liquid medium and the use of the plate count method in the order of US ≥ EZX > HW > CW > B (Figure 5). The molecular weight (Mw) of pectin plays a crucial role in determining its prebiotic activities [76]. Consistent with this, the US and EX pectins with lower Mws compared with CW and HW (Table 1) resulted in higher viable colonies of *Lactis brevis*. Mechanical and enzymatic actions can modify pectin structure, creating substrates favorable for the growth of probiotic microorganisms by breaking down larger pectin molecules into smaller, more soluble fragments [61]. This corresponds with findings indicating that ultrasound- and enzyme-modified low-molecular-weight pectin stimulates the growth of lactic acid bacteria and bifidobacteria, which exhibit better prebiotic activity than unmodified pectin [77,78]. This study highlights the potential of dragon fruit peel pectins, particularly those from the US and EZX extraction methods, in promoting *Lactis brevis* proliferation, suggesting their promising candidacy for use as natural food prebiotics.

## 4. Conclusions

This study investigated various extraction methods for obtaining pectin from dragon fruit peel and presented a structure–function relationship to provide insights into obtaining customized pectin for specific food applications. The extraction methods significantly influenced pectin yield, molecular weight, and functional properties. Cold-water and hot-water extraction yielded pectins with higher molecular weights (1.01–1.21 × 10^3^ kDa), viscosity, and enhanced emulsifying properties (>70%), making them suitable for thickening and emulsifying applications in the food industry. In contrast, the ultrasound- and enzyme-assisted methods produced pectins with reduced molecular weights (US: 0.97 × 10^3^ kDa; EZX: 0.84 × 10^3^ kDa), correlating with improved functional properties such as enhanced antioxidant (21.54–61.11%; 27.05–74.29%) and prebiotic functions. Moreover, the higher pectin yield of EZX (20.22%) corresponded to its reduced molecular weight. Structural analysis confirmed that the pectin structure remained unchanged across extraction methods. However, different extraction methods could be employed to achieve customizable functional properties based on intended food applications. For instance, cold-water-extracted pectin has higher emulsifying properties and emulsion stability, which can be utilized as a source of natural emulsifiers. EZX-extracted pectin exhibited substantial proliferation effects on *Lactobacillus brevis* and has the potential to be used as a prebiotic source. Overall, our findings provide fundamental insights into the impact of extraction methods on dragon fruit peel pectin functionalities. Future studies could explore the optimization of the enzymatic extraction of dragon fruit peel pectin and its application as a functional ingredient in commercial food matrices.

## Figures and Tables

**Table 1:** Physicochemical properties of pectin.

	CW	HW	US	EZX
Yield (%)	10.93 ± 1.21 ^c^	15.03 ± 1.44 ^b^	15.17 ± 0.75 ^b^	20.22 ± 1.75 ^a^
Protein (%)	3.97 ± 0.05 ^c^	4.17 ± 0.03 ^b^	4.56 ± 0.09 ^a^	3.37 ± 0.06 ^d^
DE (%)	50.88 ± 0.27 ^a^	46.82 ± 0.61 ^b^	51.79 ± 0.13 ^a^	47.74 ± 0.70 ^b^
Mw (×10^3^ kDa)	1.21 ± 0.12 ^a^	1.01 ± 0.10 ^a^	0.97 ± 0.09 ^a^	0.84 ± 0.03 ^b^
Mw/Mn	4.81	4.19	4.22	13.04
L*	65.00 ± 0.10 ^b^	64.10 ± 0.80 ^bc^	63.00 ± 0.45 ^c^	69.08 ± 0.88 ^a^
a*	20.06 ± 0.24 ^a^	17.01 ± 0.77 ^b^	19.90 ± 0.08 ^a^	9.13 ± 0.10 ^c^
b*	7.73 ± 0.04 ^b^	7.23 ± 0.32 ^b^	4.81 ± 0.22 ^c^	10.06 ± 0.16 ^a^
Betacyanin (mg/L)	50.65 ± 1.56 ^a^	31.01 ± 0.26 ^c^	43.49 ± 0.78 ^b^	20.74 ± 0.26 ^d^

DE = degree of esterification; Mw = molecular weight; L* = lightness; a* = green to red; b* = blue to yellow; CW = cold-water extraction; HW = hot-water extraction; US = ultrasound-assisted extraction; EZX = enzyme-assisted extraction (xylanase). Superscripts (a–d) indicate significant difference (*p* < 0.05).

**Table 2:** Monosaccharide composition and molar ratios of pectin.

Mol%	CW	HW	US	EZX
Man	1.21 ± 0.18 ^b^	1.27 ± 0.01 ^b^	0.59 ± 0.08 ^c^	1.63 ± 0.23 ^a^
Rha	8.76 ± 0.17 ^ab^	9.36 ± 0.15 ^a^	8.11 ± 0.07 ^b^	9.51 ± 0.47 ^a^
GalA	77.21 ± 0.42 ^c^	78.51 ± 0.35 ^b^	83.12 ± 0.31 ^a^	78.25 ± 2.26 ^bc^
Glu	5.51 ± 0.01 ^a^	3.62 ± 0.15 ^bc^	2.70 ± 0.06 ^c^	4.03 ± 0.60 ^b^
Gal	3.68 ± 0.10 ^b^	4.51 ± 0.07 ^a^	4.56 ± 0.09 ^a^	4.71 ± 0.42 ^a^
Ara	3.86 ± 0.06 ^a^	2.73 ± 0.02 ^b^	0.93 ± 0.02 ^d^	1.87 ± 0.54 ^c^
HG	68.45 ± 0.59 ^b^	69.15 ± 0.50 ^b^	75.02 ± 0.38 ^a^	68.73 ± 2.73 ^b^
RG-I	25.05 ± 0.37 ^a^	25.95 ± 0.35 ^a^	21.70 ± 0.25 ^b^	25.61 ± 1.90 ^a^
HG/RG	2.73 ± 0.06 ^b^	2.66 ± 0.05 ^b^	3.46 ± 0.06 ^a^	2.69 ± 0.31 ^b^
Rha/GalA	0.11 ± 0.00 ^a^	0.12 ± 0.00 ^a^	0.10 ± 0.00 ^b^	0.12 ± 0.01 ^a^

Man = mannose; Rha = rhamnose; GalA = galacturonic acid; Glu = glucose; Gal = galactose; Ara = arabinose; HG = homogalacturonan; RG-I = rhamnogalacturonan-I; CW = cold-water extraction; HW = hot-water extraction; US = ultrasound-assisted extraction; EZX = enzyme-assisted extraction (Xylanase). Superscripts (a–d) indicate significant difference (*p* < 0.05).

**Table 3:** Rheological behavior of pectin solutions.

Model	Parameter	Pectin Samples			
		CW	HW	US	EZX
η = *Kγ*^(*n*−1)^	*K*	2613.51	1176.96	943.42	400.41
	*n*	0.37	0.53	0.53	0.5
	*r* ^2^	1.00	1.00	0.99	0.99

*K* = flow consistency index; *n* = flow behavior index; CW = cold-water extraction; HW = hot-water extraction; US = ultrasound-assisted extraction; EZX = enzyme-assisted extraction (xylanase).

## Data Availability

The data presented in this study are available in this article.

## Nomenclature

CW	Cold-water extraction
HW	Hot-water extraction
US	Ultrasound-assisted extraction
EZX	Enzyme-assisted extraction (Xylanase)
Man	Mannose
Rha	Rhamnose
GalA	Galacturonic acid
Glu	Glucose
Gal	Galactose
Ara	Arabinose
HG	Homogalacturonan
RG-I	Rhamnogalacturonan-I
DE	Degree of esterification
Mw	Molecular weight
EC	Emulsifying capacity
ES	Emulsifying stability
